# Adherence to hypertension management at a rural hospital in Limpopo: A cross-sectional study

**DOI:** 10.4102/safp.v67i1.6136

**Published:** 2025-08-18

**Authors:** Murendeni F. Sikhau, Mbuyisa J. Makhubu, Gert J.O. Marincowitz, Clara Marincowitz

**Affiliations:** 1 Department of Family Medicine, Faculty of Health Sciences, University of Limpopo, Mankweng, South Africa; 2 Department of Biological Sciences, Faculty of Science, University of Cape Town, Cape Town, South Africa

**Keywords:** hypertension, adherence to treatment, South Africa, rural, district hospital

## Abstract

**Background:**

Non-adherence to treatment remains a major contributing factor to uncontrolled hypertension and its complications. In South Africa, an estimated 50% of adults are living with hypertension and between 41.9% and 45.5% adhere to their treatment. Knowing reasons for non-adherence, therefore, is important in the treatment of hypertension.

**Methods:**

A cross-sectional study involving 243 hypertensive patients was conducted from May to July 2022 at Voortrekker Hospital, Mokopane, Limpopo province. The questionnaire included socio-demographic and hypertension-related medical information and adherence was assessed using the previously validated Therapeutic Adherence Scale for Hypertensive Patients (TASHP).

**Results:**

Forty-two per cent of participants adhered to their antihypertensive treatment, whereas 56% controlled their blood pressure. Variables such as employment (*p* = 0.0076), secondary and tertiary education (*p* = 0.0048), duration of hypertension of less than a year (*p* = 0.019) and level of income (more than R3000/month) (*p* = 0.033) were significantly associated with better adherence.

**Conclusion:**

Adherence to treatment and blood pressure control among hypertensive patients in the Mokopane area is still inadequate, although within the same range as reported in the literature. Effective strategies must be developed to address adherence, especially for vulnerable patients.

**Contribution:**

The study identified that only 42% of patients in a rural district hospital setting adhere to their hypertension management. Furthermore, it was found that patients less educated, unemployed, having an income of less than R3000/month or living with hypertension for more than 15 years are significantly more vulnerable to poor adherence.

## Introduction

Hypertension is a major public health burden worldwide, with nearly 1.13 billion people affected. It is also a major risk factor for chronic kidney disease and cardiovascular disease, including stroke, myocardial infarction and heart failure, which are among the leading causes of global morbidity and mortality.^1,2,3,4,5^

The WHO global report on hypertension found that 54% of adults (30–79-years-old) with hypertension know they have condition, 42% are on treatment and only 21% of all hypertensives have their disease under control.^4^ There is an increased prevalence in low- and middle-income countries and this has been attributed mainly to urbanisation, an ageing population, and risk factors such as obesity and sedentary lifestyles.^6,7^ During the last four decades, there has been a decline in uncontrolled hypertension in high-income countries, with a significant increase in low- and middle-income countries (LMICs) in sub-Saharan Africa, South Asia, and central and Eastern Europe.^4,8,9,10^ In South Africa, an estimated 50% of adults are living with hypertension.^11^ A study by Woodiwiss et al. found that in South Africa 49.2% of hypertensives were receiving medication and only 57.5% of individuals receiving antihypertensive medication had controlled blood pressure (BP). The proportion of all hypertensives with controlled BP was only 28.3%.^12^

Hypertension awareness, treatment and control remain inadequate worldwide despite many advances in hypertension care.^13^ A major contributor to the inadequate control of hypertension is poor adherence.^14,15^ In Africa, non-adherence to hypertensive treatment has been reported as 62.5%.^16^ Adherence in Asian countries is similar to the estimated global non-adherence rate of 45.3%.^16,17^ Poor adherence has been attributed to socio-demographics, healthcare systems, treatment factors, people’s health conditions and patient-related factors.^5,18^

Dhar et al. found that 47.3% of people adhered to hypertension treatment in the 19 countries included in their review.^19^ Enslin et al. found in a review of South African studies an adherence rate of 41.9%.^20^ A survey conducted in the Vhembe District of Limpopo province found that 45.5% of hypertensive patients reported poor adherence.^21^

Several studies have described the importance of context-specific factors associated with adherence when planning specific interventions.^16,17,18,19,20,21^ There are, however, limited data from a rural South African context available on the prevalence and factors contributing to adherence and control of hypertension.^20,21^ The study reported here therefore aimed to assess the prevalence and factors associated with adherence to hypertensive management reported by patients attending Voortrekker Hospital, in Mokopane town, 55 km south of Polokwane, the provincial capital of Limpopo province. The hospital is situated in the Mogalakwena Local Municipality, which covers 6170.3 km^2^ with a population of 325 292 people. The municipality consists of 178 villages, Mahwelereng township with 41 072 people and Mokopane town with a population of 30 151. Sixty-one per cent of the population has a monthly income of less than R3000. The unemployment rate for the municipality is 40.2%. The municipality has 3 hospitals with 29 clinics. Voortrekker Hospital and its 9 clinics serve a population of 126 717, mostly living in rural villages and Mokopane town and Mahwelereng township.^22^

## Methods

A quantitative, cross-sectional descriptive study was conducted from May to July 2022 in the outpatient department (OPD) of Voortrekker Hospital. The sample size was calculated at 240, considering a study population of 600 hypertensive patients receiving treatment at Voortrekker Hospital using the Yamane formula with a margin of error of 5%.^23^ Consecutive, consenting adult, hypertensive patients 18 years and older, who were on one or more antihypertensive agents for at least 6 months and attended the OPD during the study period, were included until the required number of participants was reached. Those too sick to participate, or who were mentally incapable of giving consent, pregnant or on treatment for less than 6 months, were excluded. None of the patients selected to participate refused.

### Data collection

A modified, validated Therapeutic Adherence Scale for Hypertensive Patients (TASHP) questionnaire was used to collect data.^24,25^ This instrument included questions about adherence to medication and lifestyle factors such as diet, exercise, stress management, tobacco and alcohol use and also the frequency of BP monitoring. The instrument included 25 questions (see Table 1). A 5-point Likert response scale was used for each item, and reverse scores were assigned to negative questions, with a score of 5 being the most desirable measure. The maximum score was 125. A score of less than 109 was considered poor adherence and 109 or more was regarded as good. In addition to the 25 adherence questions, there was a section on demographic and medical data.

**TABLE 1:** Therapeutic adherence scale for hypertensive patients questions.

Number	Questions
**Category 1: Adherence to taking medicine**	
1	I never take alternative medication for HPT
2	I take HPT medication prescribed by doctors
3	I take HPT medication according to the prescribed doses
4	I take HPT medication according to the prescribed times
5	I take HPT medication according to the prescribed intervals
**Category 2: Poor medication behaviour**	
6	I do not forget to take my HPT medication
7	I do not need family members or friends to remind me to take HPT medication
8	I do not forget to bring HPT medication when I travel or leave home
9	I do not change the dose of HPT medication according to the level of my blood pressure
10	I do not change the dose of HPT medication according to my symptoms
11	I do not stop taking HPT medication when I feel good
12	I do not stop taking HPT medication when my blood pressure is under control
13	I do not stop taking HPT medication when my disease gets worse after taking HPT medication
**Category 3: Lifestyle modification (daily life management)**	
14	I try to reduce sodium intake
15	I try to reduce my oil intake
16	I try to increase the fresh vegetable and fruit intake
17	I insist on 3–5 times regular exercise of over 30 min per week: such as walking, jogging, playing ball, swimming, aerobics, etc.
18	I try to control my weight
19	I try to take part in more-social activities
20	When I encounter something unhappy, I look on the bright side
21	I use various methods to relieve stress, such as talking, watching TV, surfing the internet, taking deep breaths, meditating, singing, etc.
22	I try to get enough sleep
23	I check my blood pressure regularly
**Category 4: Smoking and alcohol management**	
24	I don’t smoke
25	I don’t drink

*Source:* Tang HY, Zhu JC, He HY, Qian C, Yang Y. Development and evaluation of a new therapeutic adherence scale for hypertensive patients. J Third Military Med Univ. 2011;33(13):1400–1402

HPT, hypertension; TV, television.

Besides English, a language expert translated the questionnaire into two other languages commonly used in the Mokopane area (Sepedi and Xitsonga). A pilot study tested the data collection tool on 20 patients from another nearby institution and was thereby confirmed as suitable and no changes were made to the original questionnaire. The results from the pilot study were not included in this research. Participants were individually seen by the research assistant, who was fluent in all languages spoken in the Mokopane area. After the study procedure was explained and written consent was obtained from potential participants who met the eligibility criteria, the research assistant administered the questionnaires to the participants. To improve reliability and reduce bias, the research assistant was trained to use the TASHP questionnaire with Likert scale questions. She was not a health worker employed at the hospital, which enhanced reliability and participants were encouraged to feel free to give honest answers without a fear of influencing their management at the hospital. Participants’ BP was measured using the same digital and calibrated Dinamap BP monitor, after resting the subject for at least 3 min before the measurement, who was seated with the back supported, the full arm bare and resting on a surface at the level of the heart with an appropriate cuff size.^26^ The BP reading was repeated a second time if recorded higher than 140/90. After the questionnaire was completed, patients were taken to the doctor for consultation.

### Data analysis

At the end of each day, the data were captured on an Excel spreadsheet, which were then checked and cleaned. The data included the 25 adherence questions (see Table 1), demographic data, disease information and BP readings (see Table 2). Participants with BP of 140/90 mmHg or less were considered controlled in the context of this study. Descriptive statistics of the patients’ demographic data were summarised as frequencies and percentages and presented in charts and a table. A chi-squared test was used to identify the association between demographic information and adherence to hypertensive management. A *p*-value of 0.05 or less was considered statistically significant. IBM Statistical Package for the Social Sciences (SPSS) version 28.0 was used for the analysis.

**TABLE 2:** Demographics of study population and factors of adherence.

Variable	Description	Demographic data		Poor adherence		Good adherence		*p*
		Total	%	*n*	%	*n*	%	
Age (years)	< 30	2	0.8	1	50.0	1	50.0	0.408
	30–40	11	4.5	7	63.6	4	36.4	
	41–50	22	9.1	10	45.5	12	54.5	
	51–60	56	23.0	28	50.0	28	50.0	
	> 61	152	62.6	94	61.8	58	38.2	
Gender	Female	147	60.5	83	56.5	64	43.5	0.653
	Male	96	39.5	57	59.4	39	40.6	
	Total	243	100.0	140	57.6	103	42.4	
Place of residence	Rural or village	130	53.5	78	60.0	52	40.0	0.228
	Semi-rural or township	79	32.5	47	59.5	32	40.5	
	Urban or town	34	14.0	15	44.1	19	55.9	
Marital status	Single	51	21.0	26	51.0	25	49.0	0.441
	Married	141	58.0	80	56.7	61	43.3	
	Widowed	35	14.4	25	71.4	10	28.6	
	Divorced or Separated	14	5.8	8	57.1	6	42.9	
	Living with partner	2	0.8	1	50.0	1	50.0	
Highest level of education	Never attended school	33	13.6	25	75.8	8	24.2	0.0048
	Primary school	63	25.9	43	68.3	20	31.7	
	Secondary school	111	45.7	57	51.4	54	48.6	
	Tertiary	36	14.8	15	41.7	21	58.3	
Employment	Old-age grant	155	63.8	97	62.6	58	37.4	0.0076
	Unemployed	45	18.5	29	64.4	16	35.6	
	Public sector	8	3.3	2	25.0	6	75.0	
	Private sector	25	10.3	8	32.0	17	68.0	
	Self-employed	10	4.1	4	40.0	6	60.0	
Monthly income (ZAR)	< 3000	201	82.7	123	61.2	78	38.8	0.033
	3000–10 000	27	11.1	14	51.9	13	48.1	
	11 000–20 000	11	4.5	2	18.2	9	81.8	
	21 000–39 000	3	1.2	1	33.3	2	66.7	
	≥ 40 000	1	0.4	0	0.0	1	100.0	
Access to healthcare facility (km)	< 5	35	14.4	15	42.9	20	57.1	0.279
	5–10	161	66.3	97	60.2	64	39.8	
	11–20	29	11.9	18	62.1	11	37.9	
	> 20	18	7.4	10	55.6	8	44.4	
Duration of HPT (years)	< 1	30	12.3	11	36.7	19	63.3	0.019
	1–4	43	17.7	25	58.1	18	41.9	
	5–9	61	25.1	32	52.5	29	47.5	
	10–15	47	19.3	27	57.4	20	42.6	
	> 15	62	25.5	45	72.6	17	27.4	
Duration of HPT medication (years)	> 6m – < 1	33	13.6	13	39.4	20	60.6	0.078
	1–2	23	9.5	12	52.2	11	47.8	
	3–5	49	20.2	30	61.2	19	38.8	
	6–9	34	14.0	17	50.0	17	50.0	
	> 10	104	42.8	68	65.4	36	34.6	
No. of HPT drugs	1	31	12.8	16	51.6	15	48.4	0.878
	2	122	50.2	70	57.4	52	42.6	
	3	73	30.0	44	60.3	29	39.7	
	≥ 4	17	7.0	10	58.8	7	41.2	
BP current	Controlled	137	56.4	81	59.1	56	40.9	0.588
	Uncontrolled	106	43.6	59	55.7	47	44.3	
Other medical conditions	Yes	164	67.5	91	55.5	73	44.5	0.334
	No	79	32.5	49	62.0	30	38.0	

**Total**		**243**	**100.0**	**140**	**57.6**	**103**	**42.4**	-

BP, blood pressure; HPT, hypertension; No., number.

### Ethical considerations

Ethical clearance was obtained from the Turfloop (University of Limpopo) Research and Ethics Committee on 29 March 2022 (TREC/52/2022:PG). Permission to conduct the research was then granted by the Limpopo Department of Health (LP_2023_06_004), the Waterberg District Health Authority and by the CEO of Voortrekker Hospital. No patient identifiers were used when information was captured to ensure privacy and confidentiality.

## Results

Questionnaires were completed by 243 patients, 60% of whom were female, 58% were married, 63% were older than 60 years, and 46% had secondary school as their highest level of education. Fifty-four per cent of participants resided in rural areas, and 66% had to travel 5 km – 10 km to the healthcare facility. Sixty-four per cent of participants were receiving a social grant and 83% had a monthly income of less than R3000. Forty-three per cent had been on hypertension treatment for more than 10 years, whereas 14% had received it for less than a year. Sixty-seven per cent of the participants also had other medical conditions, and 50% were taking two antihypertensive drugs (see Table 2).

Fifty-six per cent of the participants had BP of 140/90 or less and were considered controlled. Fifty-eight per cent of the participants scored less than 109/125 on the TASHP questionnaire. A score of 109 or more out of a possible 125 (see Table 1) is considered adherent.^24,25^ Cronbach’s alpha, used to measure the reliability of the questionnaire, was 0.61, indicating an acceptable level of internal consistency.

Four factors were significantly associated with poor adherence, namely, being unemployed (*p* = 0.0076), having a monthly income of less than R3000 (*p* = 0.033); having a relatively low level of education (*p* = 0.0048) and having had hypertension for more than 15 years (*p* = 0.019) (Table 2). Factors such as age, gender, place of residence, marital status, distance to care, duration of HPT treatment, number of drugs taken, BP control and comorbidities were not found to be statistically significantly associated with adherence.

## Discussion

Our study was carried out in a single public sector district hospital outpatient department serving a population from either rural villages, the township or town in Limpopo Province. We found that 58% of the participants reported inadequate adherence to their hypertensive management and 56% were found to have controlled BP. Our subjects comprised mainly rural, elderly people, on grants, who were unemployed or with inadequate minimum monthly incomes. The study population of patients accessing hypertensive care at the hospital is skewed towards patients with a little more resources, as the poorest of the poor would access care at their local clinics.

Our study also indicated that 42% of the participants reported adequately adhering to antihypertensive management. Similar trends were found in a study conducted in Saudi Arabia, where 42% of 306 patients adhered to treatment.^27^ The adherence rate in our study was slightly higher than that reported in Africa (34.1%) and elsewhere in South Africa (36.3%).^6,28^

We found that 56% of our participants had controlled BP, which was in similar range as the literature. Woodiwiss et al. found in South Africa that 57.5% of individuals receiving antihypertensive medication had controlled BP.^12^ In Sedibeng, South Africa, BP was controlled in 50.2% of the respondents.^28^ Feng et al.^29^ reported a hypertension control rate of 64.4% in rural China. Ni et al. reported a hypertensive control rate of 32.3%^30^ in Shenzhen, China. The latter is similar to the Beaney et al.’s study^31^ that assessed hypertension control in 92 countries, which found a hypertension control rate of 31.7%.^31^ There is the possibility that the population of patients coming to the hospital for care are more adherent to treatment as they have easier access than those in distant villages. Our findings indicated that 80.7% of participants were living less than 10 km from the hospital.

We found no statistically significant association between BP control and adherence. This unexpected finding could be because of the sample size and other forms of bias introduced by the convenient sampling and single facility-based study. In our study, only 41% of those with controlled and 44% of those with uncontrolled BP demonstrated good adherence. This contradictory observation could be explained by the fact that inadequate treatment regimes, the presence of complications and secondary causes of hypertension could also be contributing to this result. In a hospital-based study, more participants with complicated hypertension are expected, considering referral guidelines. Khadoura et al.^32^ also found no statistically significant association between adherence and hypertension control in their randomised control trial performed in Gaza. In contrast, Desta et al.^33^ found in a meta-analysis of studies conducted in Ethiopia that patients with good adherence are significantly less likely to have poor hypertension control.

We found a statistically significant association between adherence and level of education, income and employment status. Better-educated participants had superior adherence, as reflected in several studies.^1,17,28^ Our findings that those patients who were unemployed, on social grants or had a monthly income of less than R3000 are significantly less inclined to follow treatment, were echoed by studies conducted in Nigeria, Kenya, Ethiopia and Indonesia.^7,14,34,35^

In our study, participants who had HPT for more than 15 years were significantly less likely to maintain treatment, whereas some surveys found the opposite.^24,35^ This could be because most of our subjects who were on treatment for many years, with complicated treatment regimes, suffered treatment fatigue.^36^ It could also be that poor insight and limited knowledge about their condition, or the sheer hardship of daily living, contribute to this finding, as suggested by Hamrahian et al.^18^

We found no significant association between adherence and age, gender, place of residence, marital status, distance to care, duration of HPT medication, the number of drug treatments the participants were taking, BP control or the presence of comorbidities. The literature is inconclusive about several of these factors. Masilela et al.^37^ and Macquart de Terline et al.^38^ also found no definite association between gender and adherence. In contrast, Pan et al.^24^ found that women were significantly better at adherence. Several studies found an association between advanced age and poor adherence,^32,36,39,40^ which is contrary to Choi et al.,^41^ who found better adherence in older people. The literature generally supports the notion that distance from health facilities makes a difference to people’s adherence.^7,14^ However, Algabbani and Algabbani^27^ found that, in Saudi Arabia, distance to care facilities was not associated with better adherence to treatment.

## Conclusion

Adherence to treatment was 42% and BP control was 56% among hypertensive patients in the Mokopane area lying within the same range as reported in the literature. Research is needed to evaluate adherence and BP control at community level, as a hospital-based study could be biased towards the more adherent patients. More research is also needed to understand the lack of correlation between treatment adherence and BP control.

Because most patients attending public health facilities in South Africa are unemployed, on a social grant, or with a monthly income below R3000, poor adherence is a serious threat to effective patient care in our setting. A concerted effort should be made to care for these vulnerable patients to improve their adherence to treatment and enhance control of their hypertension. Hamrahian et al.^18^ suggested that the secret to improved adherence to treatment lies in patient-centred, respectful and compassionate health worker–patient relationships, in addition to sustained efforts to improve patients’ understanding of their condition and involvement in their care through personalised BP monitoring.^18^

## Limitations

This study has limitations, as do all cross-sectional surveys. It was carried out at a single institution and thus the results are not generalisable. We used consecutive sampling, which is a weak way of selecting a study population, and included only hypertensive patients who were available at the time our subjects were selected. Self-reported questionnaires are susceptible to recall bias by either over-reporting or under-reporting past health behaviour or inability to remember past medical information. Other factors recorded in the literature to influence adherence, such as the health system our patients experienced, patient–doctor relationships, self-efficacy, social support and self-care, were not explored.
